# Diosmin Alleviates Venous Injury and Muscle Damage in a Mouse Model of Iliac Vein Stenosis

**DOI:** 10.3389/fcvm.2021.785554

**Published:** 2022-01-13

**Authors:** Zhiye Guo, Xiaolong Du, Yihua Zhang, Chunwan Su, Feng Ran, Qiulun Lu

**Affiliations:** ^1^Key Laboratory of Cardiovascular and Cerebrovascular Medicine, Collaborative Innovation Center for Cardiovascular Disease Translational Medicine, Nanjing Medical University, Nanjing, China; ^2^Department of Vascular Surgery, Nanjing Drum Tower Hospital, The Affiliated Hospital of Nanjing University Medical School, Nanjing, China

**Keywords:** iliac vein stenosis, venous hypertension, venous permeability, endothelial integrity, diosmin

## Abstract

Chronic venous disease (CVD) is a progressive inflammatory disease that increases in prevalence with age. Elucidating the underlying molecular mechanism of CVD development is essential for disease prevention and treatment. This study constructed a mouse model of iliac vein stenosis to explore the mechanism of the CVD disease progression, and diosmin was administered as a positive control (as recommended by clinical practice). The mouse model was established successfully with iliac vein stenosis, leading to the expansion of the intercellular space and venous leakage. Conversely, micronized diosmin showed a dose-dependent therapeutic effect for these manifestations. Concerning the mechanism, iliac vein stenosis caused an inflammatory response in veins, while diosmin suppressed this increase. Furthermore, RNA sequencing analysis indicated that diosmin significantly improved muscle function through actin filament organization and muscle contraction. These results indicated that the mouse model of iliac vein stenosis is a reliable model to study venous diseases. Furthermore, the dose-dependent therapeutic effect of diosmin on stenosis (without toxic side-effects) suggests greater protection against venous diseases at higher doses of diosmin.

## Introduction

Iliac vein stenosis (IVS) is a clinical condition resulting from external compression of the iliocaval venous unit with the consequent remodeling of the vascular wall, hemodynamic alterations, and a pre-disposition to venous thrombosis (1). IVS is a frequent pathologic process in advanced chronic venous disease (2). This pulsatile chronic venous compression stimulates the formation of fibrous adhesions, leading to partial or complete iliac vein occlusion over time (3, 4). The true prevalence of iliac vein compression syndrome is not known since the majority of patients are asymptomatic. It is estimated that iliac vein compression is present in 2–5% of patients with venous disease of the lower limbs (5). However, iliac vein compression can be symptomatic when severe, as with the formation of blood clots known as deep vein thrombosis (DVT) and pulmonary embolism (6). In addition, long-term iliac vein compression not only causes venous reflux disturbance and venous hypertension of the lower limbs, but it is also one of the reasons for venous valvar insufficiency and superficial varicose veins of the lower limbs (7–9).

Venous hypertension caused by structural or functional abnormalities of veins is a pathophysiological feature of chronic venous disease, which can cause a series of pathophysiological changes. These include endothelial dysfunction and inflammation resulting in microcirculatory and tissue damage, and ultimately varicose veins, and venous ulcers (10). Specifically, altered venous flow results in abnormal shear stress in the vein that triggers initial inflammatory responses, endothelial cell activation, as well as leukocyte adherence, activation, and infiltration. Furthermore, endothelial permeability increases red blood cell extravasation into the surrounding tissues over time (11). According to the law of hemodynamics, venous hypertension affects the microcirculatory system (including venules and capillaries), resulting in microcirculatory stasis followed by capillary failure with excessive dilatation (capillary halo formation), increased permeability and fluid filtration, swelling of the connective tissue, and high interstitial pressure (12). Despite what is known regarding the physiological processes in venous hypertension, the molecular mechanism underlying the process of IVS remains unclear.

Currently, diosmin is recommended and widely used for treatment with IVS (13). The protective effect of diosmin was explored recently in arterial models or arteriovenous fistula models (14, 15). However, the molecular mechanism underlying how diosmin protects the venous function is not explored clearly. Moreover, although the beneficial effects of diosmin can be observed in clinical practice, it has numerous commercial types, each with a broad range of particle diameters. Also, it has been shown that due to its insolubility in water, its micronized form is easier to digest and absorb (16). Taken together, we must identify the molecular mechanism underlying how micronized diosmin protects against venous dysfunction in an IVS model.

The present study is designed to verify the therapeutic effect of diosmin in an IVS model and to explore the molecular mechanism underlying how micronized diosmin protects venous function against IVS pathology. In addition, we evaluate the effects of micronized diosmin on the permeability of veins in a dose-dependent manner. This study shows how diosmin can maintain the integrity of veins in a dose-dependent manner and provides insight for developing anti-venous hypertensive therapies.

## Materials and Methods

### Mice

All animal care and experiments were approved by the Animal Care Committee of Nanjing Medical University (approval numbers NJMU-2102034). Efforts have been made to minimize the unnecessary use and discomfort of animals. The SPF C57BL/6 mice (8 weeks old) were purchased from the Animal Center of Nanjing Medical University (Nanjing, China).

### Mouse Model of IVS

The established flow restriction model of IVS was performed as described (17). Mice were anesthetized by a 1–1.5% gas isoflurane-mixture in a supine position via a nose cone. After a groin incision, vein ligatures were performed by separating the muscle tissue. The left external iliac vein was gently separated from the left external iliac artery. To achieve the stenosis, the left external iliac vein was ligated over a 30-gauge needle. A 7-0 polypropylene suture was tied over the vein and needle, before removing the needle. The skin was finally closed by a 5-0 suture. The same vascular dissections were performed without vein stenosis in sham groups. All mice were divided into five groups, including sham/normal saline (NS; *n* = 4), sham/diosmin (40 mg/kg; *n* = 4), IVS/NS (*n* = 5), IVS/diosmin (20 mg/kg; *n* = 6), and IVS/diosmin (40 mg/kg; *n* = 8). All groups received intragastric administration once a day. The mice were treated with 20 or 40 mg/kg diosmin per day (Diosmin, NMPN H20058471; Chia Tai Tianqing Pharmaceutical, Nanjing, China).

### Laser Speckle Images

Repeated measurements of hindlimb blood flow perfusion over the region of interest were obtained, ensuring that the scanning area covered both the hindlimb paws. Serial color-coded perfusion images were collected immediately after surgery (post), as well as 14 and 28 days after surgery using a laser speckle imaging system (moor FLPI-2; Moor Instruments, Axminster, UK) (**Figure 2A**). The perfusion parameters are expressed as the ratio of the left (stenosis) to the right (normal) limb (18).

### Laser Particle Size Analyzer Detection

A sample pump was used to circulate ultrapure water in a sample cup, tube, and cell, while an ultrasonic disperser was used for degassing. The injection pump parameters were kept at 2,000 rpm, while the ultrasonic power was kept at 5.0. Then, the diosmin was weighed off into the sample cup for ultrasonic treatment. The instrument shading settings were kept within the range of 8.00–12.00%, and a laser particle size analyzer (Mastersizer 2000; Panalytical, Malvern, UK) was used to detect the diameter of the diosmin particles.

### Evans Blue

Mouse retinal intravenous injection of 0.5% Evans blue stain solution (E2129; Sigma-Aldrich, St. Louis, MO, USA) was used to make the skin and soles appear blue. After 1 h, the mice were sacrificed, and the semi-membrane (SM) muscle tissue was taken out. The amount of Evans blue staining solution is determined according to the weight of the mouse. The muscle tissue was weighed, respectively, before adding formamide (F810079; Macklin, Shanghai, China), and incubated for 24–48 h in a 55°C water bath to extract Evans Blue. After centrifugation, the supernatant was measured at an optical density (OD) of 620 nm. Simultaneously, the OD values of the standard Evans blue with different known gradients were measured to draw a standard curve from which the muscle tissue content could be derived.

### Fluorescent Staining

The mouse gastrocnemius muscle was fixed in 4% paraformaldehyde (#158127; Sigma-Aldrich) overnight at 4°C. The tissues were embedded in opti-mum cutting temperature compound (OCT) (#4583; Sakura, Torrance, CA, USA), before incubating frozen sections of 5 μm with 5% goat serum, blocked for 1 h, and immunostaining with Anti-ZO1 tight junction (#61-7300; Invitrogen, Waltham, MA, USA) overnight. The sections were then incubated with CoraLite488-conjugated Affinipure Goat Anti-Rabbit IgG (H+L) for 1 h and were nuclear-stained with DAPI (P0131; Beyotime, Shanghai, China). A representative picture was taken with confocal microscopy (LSM710; Carl Zeiss, Oberkochen, Germany) at 400 × magnification.

### RNA Sequencing

The muscle tissue was collected and quickly frozen in liquid nitrogen, and then treated with TRIzol to extract total RNA. To ensure that the RNA is not degraded or contaminated, a spectrophotometer (Implen, Westlake Village, CA, USA) was used to check the purity and concentration of the RNA. Next, the Agilent Bioanalyzer instrument (Agilent Technologies, Santa Clara, CA, USA) was used to assess RNA integrity. RNA sequencing and GO analysis were performed by Frasergen Genomic Medicine (Wuhan, China).

### Real-Time PCR Assay

RNAiso plus (Takara, Shiga, Japan) was used to isolate the total RNA from the semi-membranous muscles following the manufacturer's instructions. Briefly, 1 μg RNA was used for reverse transcription using HiScript® II Q RT SuperMix kit (Vazyme, Nanjing, China). The gene-specific primers used are listed in Supplementary Table 2. The qPCR was conducted with AceQ qPCR SYBR Green Master Mix (Vazyme). The amplification reaction was performed on the StepOne™ Real-Time PCR System (Life Technologies, Carlsbad, CA, USA). The 2-ΔΔCt method was used to quantify the relative mRNA levels and normalize them to the average of the control group (shown as the average multiple of the control on the Y-axis).

### ELISA Assay

ELISA kits for mouse ICAM-1 (ab252355; Abcam, Cambridge, UK), VCAM-1 (ab201278; Abcam), alanine aminotransferase (ALT, ab282882; Abcam), aspartate aminotransferase (AST, ab263882; Abcam), and creatinine (CREA, KT21312; MSK Bio, Hyderabad, India) were used to determine the expression levels of ICAM-1, VCAM-1, ALT, AST, and CREA in the serum. The standard concentration range of the sample was linear between 5 and 1,000 pg/ml. Each serum sample was tested twice, and the average value was analyzed using GraphPad Prism v8.3.0.

### Statistical Analysis

All studies were designed to use randomization and blinded analysis to generate groups of the same size. The “n” represents the number of mice (*in vivo*). The results are expressed as mean ± standard deviation (SD), and statistical analysis was performed using the GraphPad Prism v8.3.0. The Shapiro-Wilk test was used to check the normality of the data, and the Brown-Forsythe test for equal variances. For comparisons between two or more groups, one-way ANOVA, followed by Tukey's multiple comparison test, was used. Otherwise, the Welch analysis of variance, followed by Tamhane's T2 method for *post-hoc* analysis, was used. The *pos -hoc* test was only performed when F in the ANOVA (or equivalent) reaches a statistical significance below 0.05 with no significant variance in heterogeneity. A probability value of *p* < 0.05 was considered significant.

## Results

### Detection of Diosmin Particle Diameters

**Figure 1:**
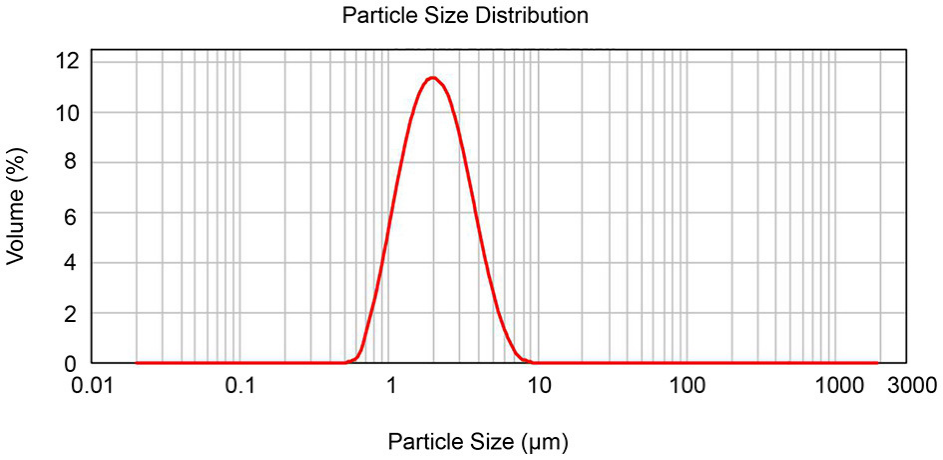
Determination of diosmin particle diameter by laser particle size analyzer. The diameter size distribution of the diosmin used in this study is presented.

Due to its difficult solubility and absorbency, the micronization of diosmin has been optimized for adoption in clinics (16). In this study, the diameters for diosmin particles were analyzed, showing a mean diameter of 2 μm and sizes of <2.9 μm for more than 80% of particles (Figure 1 and Supplementary Table 1), confirming micronization of the diosmin particles.

### Diosmin Inhibits Venous Leakage in a Dose-Dependent Manner

**Figure 2:**
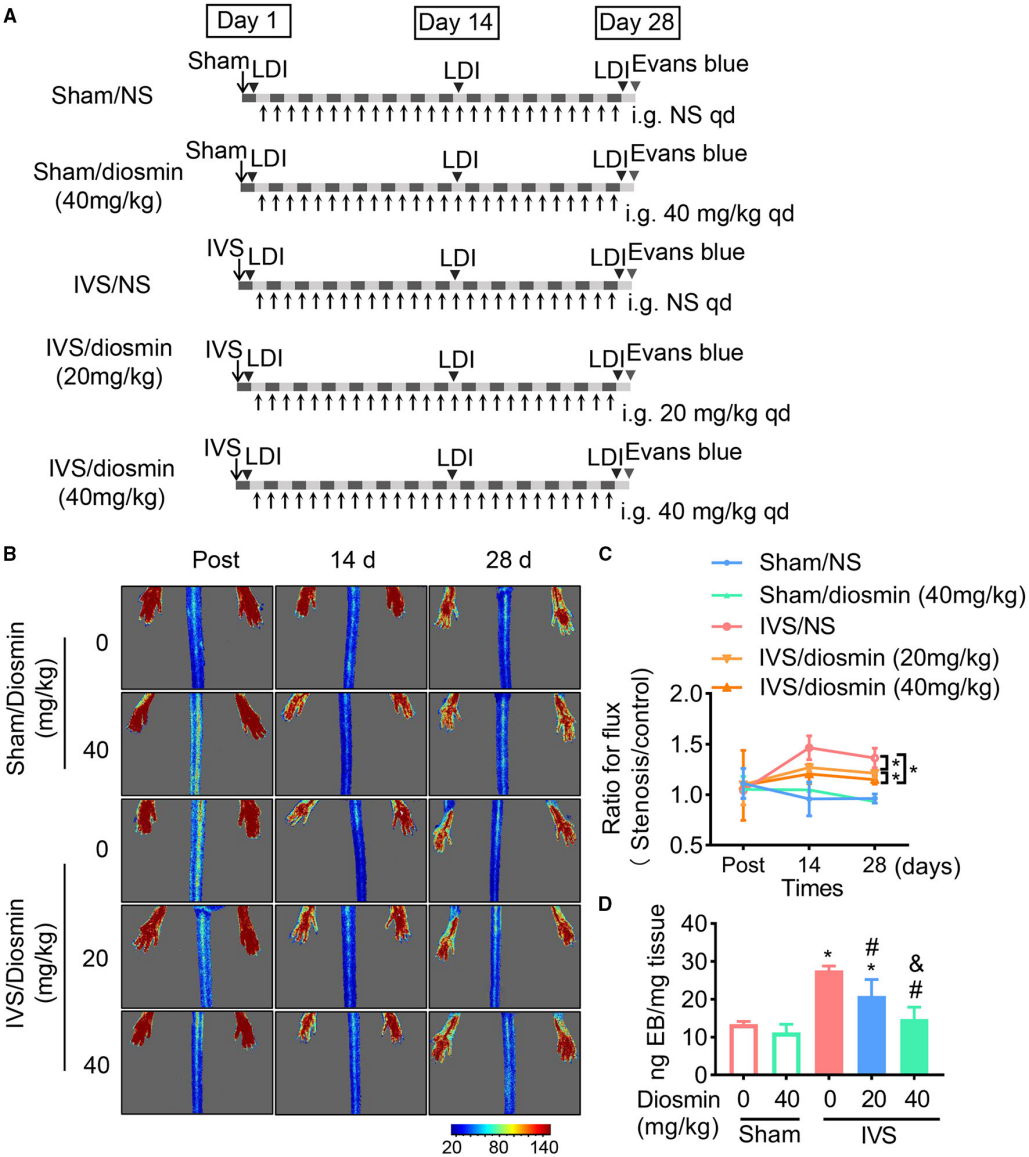
Diosmin alleviates venous permeability in a mouse model of iliac vein stenosis (IVS). **(A)** Schema of experimental procedure. **(B,C)** Blood flow parameters of mice at different time points after surgery using the laser speckle system. Sham/normal saline (NS, *n* = 4), Sham/diosmin (40 mg/kg; *n* = 4), IVS/NS (*n* = 5), IVS/diosmin (20 mg/kg; *n* = 6), and IVS/diosmin (40 mg/kg; *n* = 8). **(B)** Representative images. **(C)** The quantification analysis for laser speckle images. **(D)** The content of Evans blue in semi-membrane (SM) muscle of mice: Sham/NS (*n* = 4), Sham/diosmin (40 mg/kg; *n* = 4), IVS/NS (*n* = 5), IVS/diosmin (20 mg/kg; *n* = 6), and IVS/diosmin (40 mg/kg; *n* = 6). *p < 0.05 vs. Sham/NS; #*p* < 0.05 vs. IVS/NS; &*p* < 0.05 vs. IVS/diosmin (20 mg/kg).

To confirm the protective effect of diosmin treatment in IVS *in vivo*, we generated a mouse model of femoral vein stenosis by administering different doses of diosmin. The blood perfusion parameters were detected with a laser speckle imaging system. We found no changes under physiological conditions even when high doses of diosmin were administered (Figures 2B,C). Furthermore, diosmin did not affect the blood perfusion parameters at the early stage of IVS (Figure 2B). At 14 and 28 days, after surgery, diosmin decreased the bilateral perfusion ratio. Both the doses of diosmin at 20 and 40 mg/kg/day eliminated the increase of the perfusion ratio caused by the vein stenosis, whereas administration of the higher dose of diosmin presented a more protective effect (Figures 2B,C).

Consistent with the results from the laser speckle images, the content of Evans blue in the IVS group was increased compared to the sham group, indicating that the blood stasis resulted in an endothelial injury in the IVS model. Moreover, the higher dose diosmin (40 mg/kg/day) presented a more pronounced protective effect of vascular permeability, compared with the 20 mg/kg/day of the diosmin group (Figure 2D). These results show that diosmin inhibits venous leakage in a dose-dependent manner.

### Diosmin Maintains Endothelial Integrity in Veins

**Figure 3:**
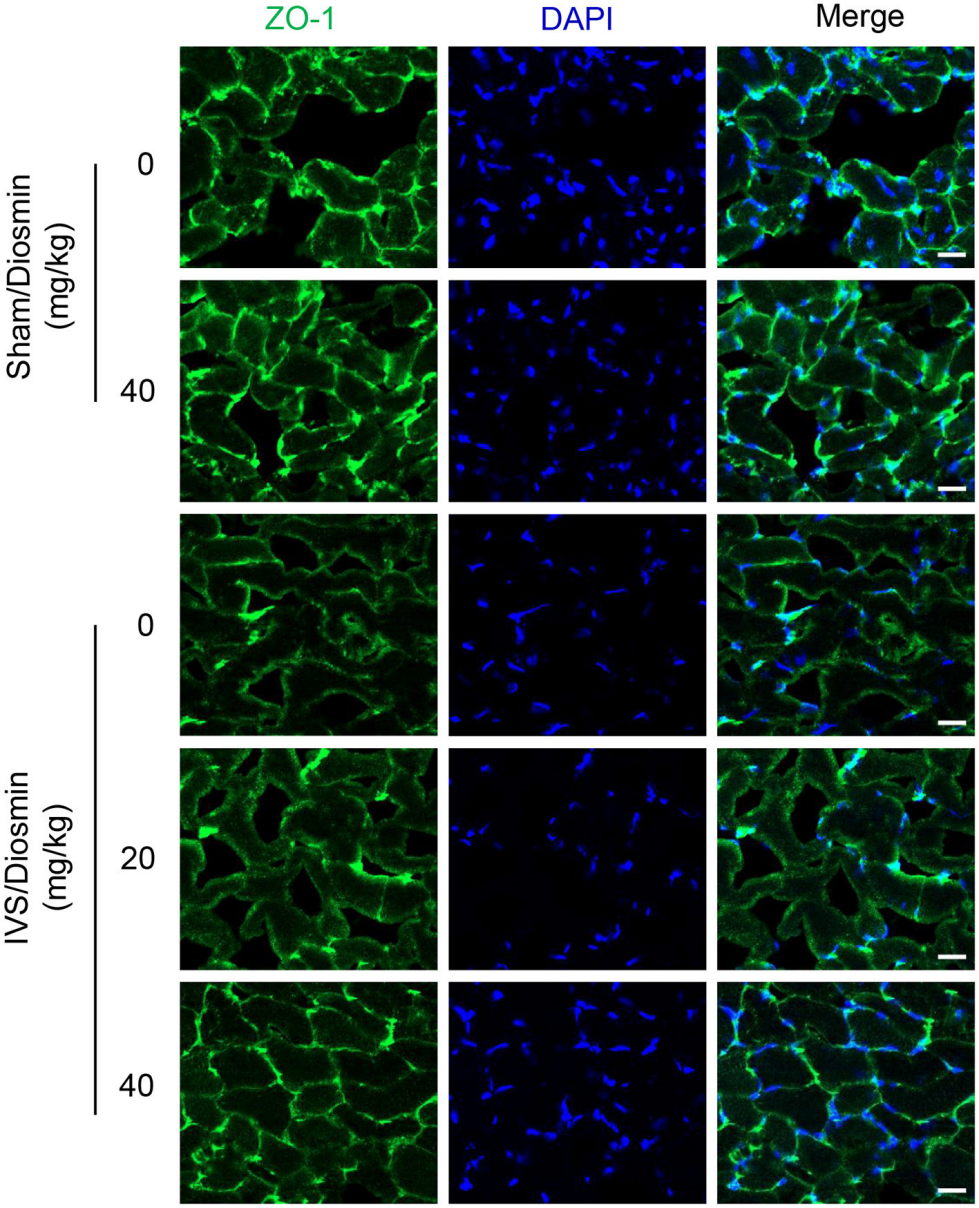
Diosmin inhibits endothelial dysfunction in a mouse model of iliac vein stenosis. Representative immunofluorescence staining images showing the change of ZO-1 at the gastrocnemius muscle cell junction.

To further confirm the protective effect of diosmin on vascular leakage, the tight junctions of cell connections were detected with immunofluorescence staining using an antibody against the Zonula occludens protein-1 (ZO-1). The gastrocnemius muscle close to the ligature sites was stained with ZO-1. We found that administration of diosmin did not affect the connections between the cells under baseline conditions (Figure 3). After 28 days of venous ligation, the fluorescence intensity at the junction of the gastrocnemius muscle bundles in the ligation group was weakened, and the morphology of the muscle bundles was incomplete, as compared with the sham group. While in the IVS/diosmin (20 mg/kg) group, the connection between the muscle bundles was improved, and the fluorescence intensity of the connection was increased. Furthermore, administration of the higher dose of diosmin at 40 mg/kg/day alleviated the expansion of the intercellular gap compared to the group with the lower dose treatment (Figure 3). These results indicate that in the IVS model, the intercellular connections of muscle cells are destroyed, and the administration of diosmin maintains the cell-cell connection and the junction in a dose-dependent manner.

### Diosmin Suppresses Inflammatory Responses in IVS

**Figure 4:**
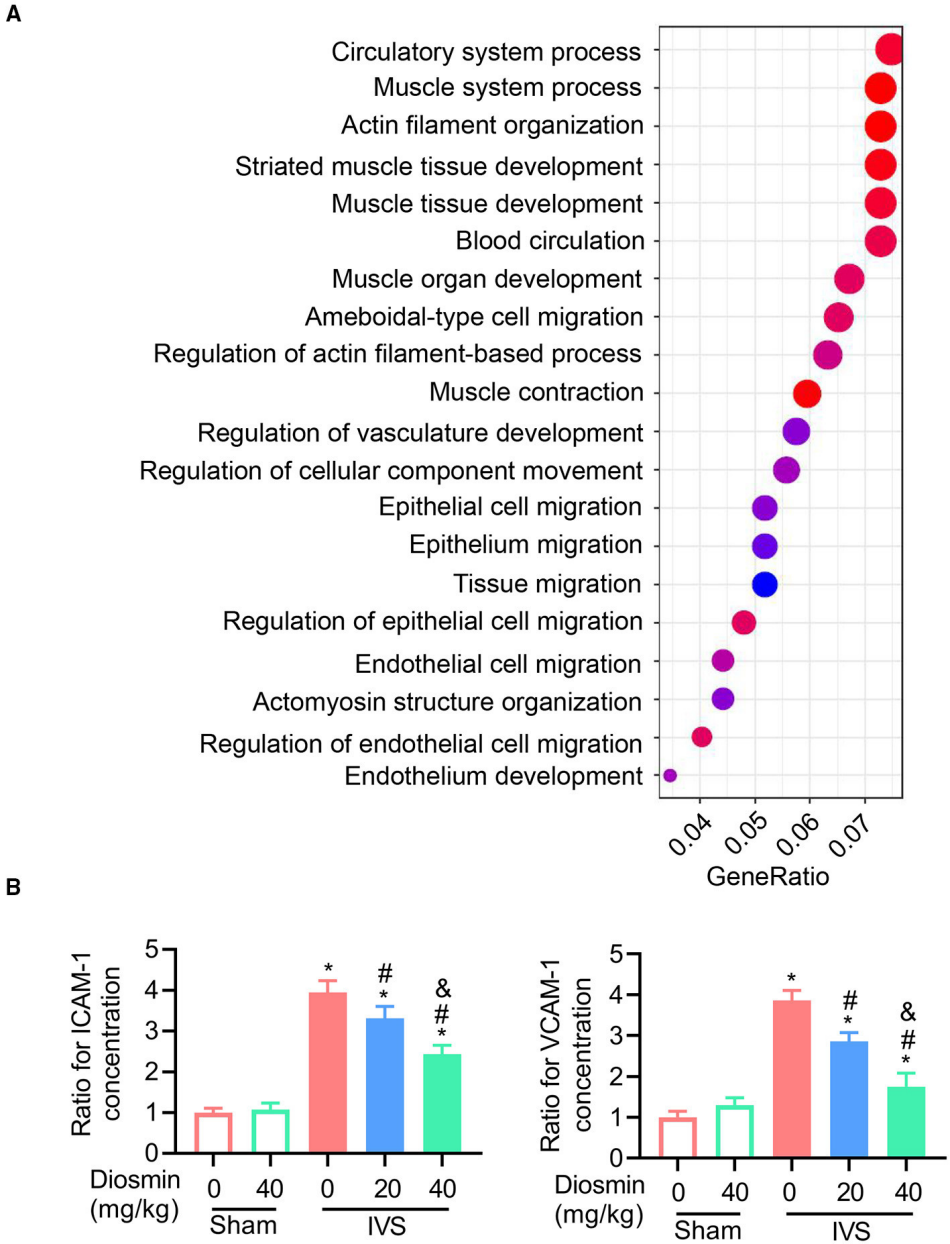
Diosmin maintains muscle function in a mouse model of iliac vein stenosis. **(A)** Gene ontology (GO) enrichment for the regulated genes in the surrounding muscles between the IVS/NS and IVS/diosmin (40 mg/kg) groups; *n* = 4 per group. **(B)** The relative protein levels of ICAM-1 and VCAM-1 in the surrounding muscles among the different groups: Sham/NS (*n* = 4), Sham/diosmin (40 mg/kg; *n* = 4), IVS/NS (*n* = 5), IVS/diosmin (20 mg/kg; *n* = 6), and IVS/diosmin (40 mg/kg; *n* = 8). *p < 0.05 vs. Sham/NS; #*p* < 0.05 vs. IVS/NS; &*p* < 0.05 vs. IVS/diosmin (20 mg/kg).

According to previous reports, diosmin suppresses the inflammatory response in pathological conditions (19, 20). In this study, we performed an ELISA assay to detect the expressions of ICAM-1 and VCAM-1 in the serum. Under physiological conditions, diosmin did not affect the levels of ICAM-1 and VCAM-1. In the mouse model of IVS, diosmin suppressed stenosis-induced increases for ICAM-1 and VCAM-1 levels, and a significant improvement in the adhesion molecule profile was also observed after a high-dose treatment of diosmin (Figure 4B).

### Diosmin Maintains the Contraction of Surrounding Muscles in IVS

**Figure 5:**
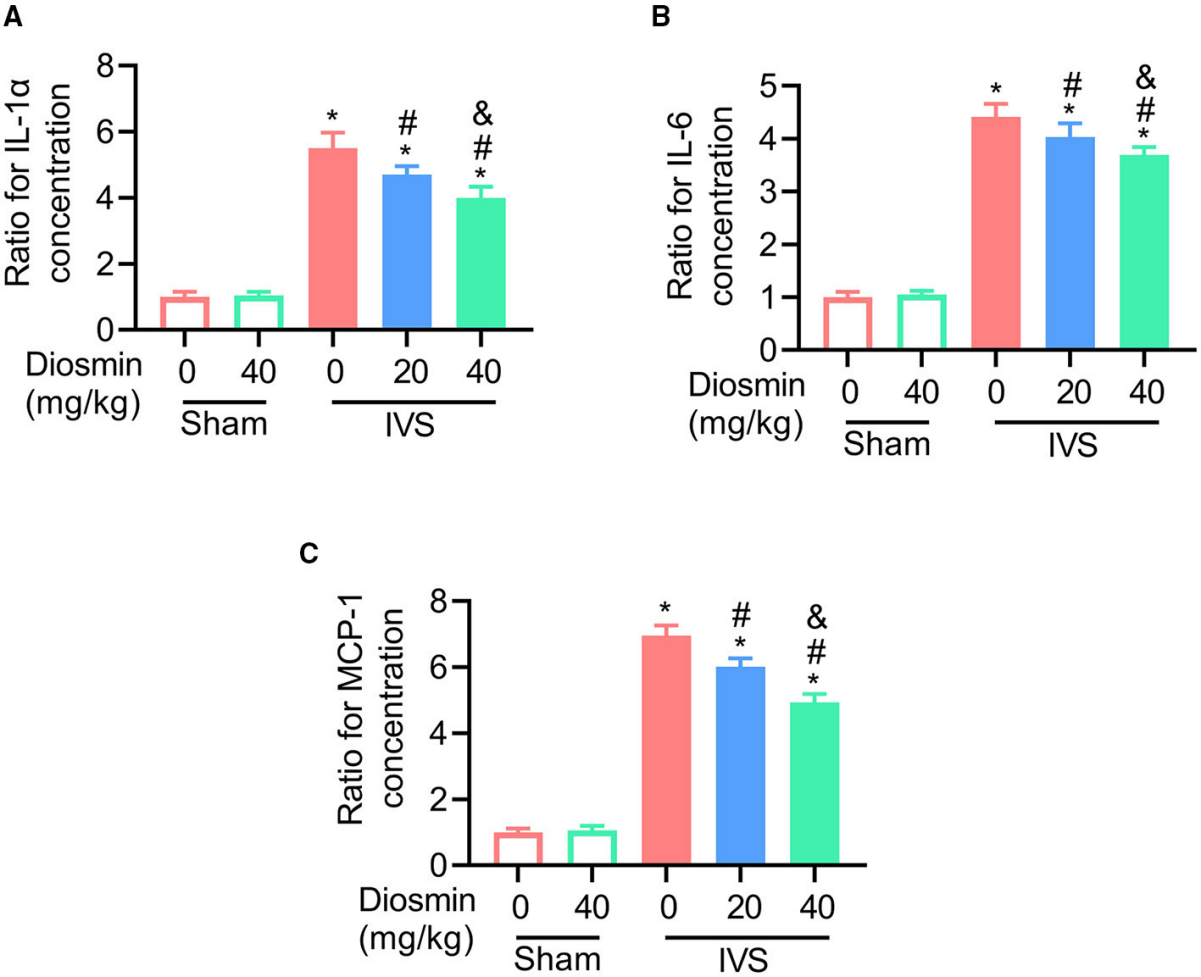
Diosmin inhibits the expression of inflammatory factors in a mouse model of iliac vein stenosis. **(A–C)** Quantified images showing the mRNA levels of IL-1α **(A)**, IL-6 **(B)**, and MCP1 **(C)**. Sham/NS (*n* = 4), Sham/diosmin (40 mg/kg; *n* = 4), IVS/NS (*n* = 5), IVS/diosmin (20 mg/kg; *n* = 6), and IVS/diosmin (40 mg/kg; *n* = 8). *p < 0.05 vs. Sham/NS; #*p* < 0.05 vs. IVS/NS; &*p* < 0.05 vs. IVS/diosmin (20 mg/kg).

To explore the cellular effects and mechanism of diosmin, RNA sequencing was performed using the semi-membranous muscle surrounding the ligated veins. GO analysis indicated that diosmin significantly maintained the function of affected muscles involved in actin filament organization and muscle contraction (Figure 4A). In addition, we further explored the expression of inflammatory factors in the semi-membranous muscles, such as IL-1, IL-6, and MCP-1. The real-time PCR assay showed that stenosis induced the expression of the inflammatory factors IL-1, IL-6, and MCP-1 mRNA. In contrast, diosmin repressed the stenosis-induced increase for these inflammation-related mRNA in muscles (Figures 5A–C).

### Diosmin Does Not Cause Kidney or Liver Injury

Although the high dose of diosmin has more powerful effects on the maintenance of venous and muscle function, it is unclear whether the high dose of diosmin at 40 mg/kg/day might cause kidney and/or liver injury. Therefore, we monitored the levels of ALT and AST at the end of the *in vivo* experiment. Our results show that the high dose of diosmin did not cause liver injury (Supplementary Figures 1A,B). Additionally, the level of CREA was not changed after the diosmin treatment (Supplementary Figure 1C). These results confirmed that diosmin does not cause kidney or liver injury at doses as high as 40 mg/kg/day.

## Discussion

Diosmin is a well-known clinically used veno-active drug in the treatment of chronic venous disease and hemorrhoidal disease. This study verified the dose-dependent therapeutic effect of micronized diosmin in an IVS model. We found that micronized diosmin can effectively repress inflammatory stress, maintain venous integrity, and alleviate the injury of vein-closed tissues.

Micronized diosmin effectively prevents venous permeability in a mouse model of IVS. During the pathophysiological condition of IVS, a dysfunctional venous system follows the venous wall and the valvar damage, incurring substantial venous permeability. Over the past decade, two main models for permeability were raised to address either the formation of trans-endothelial channels or the endothelial junctions. Extravasation of inflammatory and immune cells serves specific purposes in pathological conditions. Although these factors and cells are a prerequisite for the healing of acute diseases, they may simultaneously interfere with recovery to propagate chronic disease. In hamster models of iliac ligature, venous pressure is increased and the number of rolling leukocytes are elevated after ligation, indicative of an inflammatory response in this pathological condition (17). Moreover, in a rat model of femoral arteriovenous fistula, an increase in granulocyte and macrophage infiltration in the vascular wall has been observed, showing that inflammatory stress accompanies venous hypertension and limb edema (14). Furthermore, in venous endothelial cells, the high expression of the adhesion molecules ICAM-1 and VCAM-1 upregulate the expression of the downstream inflammatory factors TNF-α, IL-1β, and IL-6 (21). In our mouse model of IVS, we observed similar results. The micronized diosmin treatment abolished the related inflammatory stress to maintain the venous integrity, as shown by the lower level of Evans blue content. Interestingly, a high dose of micronized diosmin was more effective at alleviating venous permeability, indicating that micronized diosmin has a dose-dependent venous protective effect. These data indicate that micronized diosmin can effectively suppress the inflammatory response and maintain the venous integrity to alleviate the venous permeability in IVS.

Once venous permeability occurs, the surrounding tissues can be damaged because of the inflammatory stress in venous hypertensive pathologies. Although it is clear if permeability to solute molecules and cells is variably regulated to some extent, the tissues near the injured veins can also be impaired by these factors. The major damage is caused by certain molecules, including growth factors and inflammatory cytokines, which cross the venous walls into the surrounding tissues after the venous leakage from the increased permeability. It is known that growth factors, such as VEGF, placenta growth factor, and fibroblast growth factor, can be secreted into the nearby tissues (22–24). In a hamster model, induction of ischemia-reperfusion injury elicited a significant increase in leukocyte rolling adhesion, accompanied by enhanced leakage of FITC-dextran of 150 kDa into the perivascular tissue (14). Cell studies also showed that infiltration of granulocytes, macrophages, and lymphocytes into the surrounding muscles and tissues resulted in impaired muscle or tissue structure and function (25). In rats, femoral arteriovenous fistula caused granulocyte and macrophage infiltration into the venous wall and the surrounding tissue, as well as a lesser increase in T and B-lymphocyte infiltration (14). This infiltration was accompanied by a local increase in the proteolytic enzymes MMP-2 and MMP-9. In this study, we propose that the inflammatory stress was triggered in response to IVS, thereby impairing the nearby muscles. Conversely, micronized diosmin could prevent inflammation-induced tissue damage in a dose-dependent manner.

In conclusion, micronized diosmin administration alleviates venous permeability and tissue injury in a dose-dependent manner in IVS. Micronized diosmin abolishes inflammatory stress in response to vein stenosis and retains the maintenance of venous integrity. These findings represent the dose-dependent therapeutic role of micronized diosmin against vein stenosis to maintain the integrity and the function of the veins.

## Data Availability Statement

The original contributions presented in the study are included in the article/Supplementary Material, further inquiries can be directed to the corresponding author/s.

## Ethics Statement

The animal study was reviewed and approved by Animal Care Committee of Nanjing Medical University.

## Author Contributions

QL and FR designed the study. ZG and XD conducted the research. YZ and CS analyzed the data. QL, ZG, and YZ wrote the manuscript. All authors contributed to the article and approved the submitted version.

## Funding

This project was supported by grants from the National Natural Science Foundation of China (#81970414 to QL) and the Natural Science Foundation of the Jiangsu Higher Education Institutions of China (#19KJA350001 to QL).

## Conflict of Interest

The authors declare that the research was conducted in the absence of any commercial or financial relationships that could be construed as a potential conflict of interest.

## Publisher's Note

All claims expressed in this article are solely those of the authors and do not necessarily represent those of their affiliated organizations, or those of the publisher, the editors and the reviewers. Any product that may be evaluated in this article, or claim that may be made by its manufacturer, is not guaranteed or endorsed by the publisher.

## Abbreviations

ALT: alanine aminotransferase
ANOVA: one-way analysis of variance
AST: aspartate aminotransferase
CREA: creatinine
DVT: deep vein thrombosis
IVS: iliac vein stenosis
NS: normal saline
OD: optical density
SD: standard deviation
SM: semi-membrane
ZO-1: Zonula occludens protein-1.

## References

[1] Kutsenko O McColgan Y Salazar G. Iliac vein stenosis: is the data strong enough for stenting in the young Pelvic Venous Disorders (PeVD) Population? Tech Vasc Interv Radiol. (2021) 24:100733. 10.1016/j.tvir.2021.100733 34147201

[2] Raju S Walker W Noel C Kuykendall R Jayaraj A. The two-segment caliber method of diagnosing iliac vein stenosis on routine computed tomography with contrast enhancement. J Vasc Surg Venous Lymphat Disord. (2020) 8:970–7. 10.1016/j.jvsv.2020.02.021 32414674

[3] McDermott S Oliveira G Ergul E Brazeau N Wicky S Oklu R. May-Thurner syndrome: can it be diagnosed by a single MR venography study? Diagn Interv Radiol. (2013) 19:44–8. 10.4261/1305-3825.DIR.5939-12.1 22801870

[4] Heijmen RH Bollen TL Duyndam DA Overtoom TT Van Den Berg JC Moll FL. Endovascular venous stenting in May-Thurner syndrome. J Cardiovasc Surg. (2001) 42:83–7. 11292912

[5] Kalu S Shah P Natarajan A Nwankwo N Mustafa U Hussain N. May-thurner syndrome: a case report and review of the literature. Case Rep Vasc Med. (2013) 2013:740182. 10.1155/2013/740182 23509664 PMC3590570

[6] Evans NS Ratchford EV. Vascular disease patient information page: venous thromboembolism (deep vein thrombosis and pulmonary embolism). Vasc Med. (2014) 19:148–50. 10.1177/1358863X14529007 24829313

[7] Cockett FB Thomas ML. The iliac compression syndrome. Br J Surg. (1965) 52:816–21. 10.1002/bjs.1800521028 5828716

[8] Ibrahim W Al Safran Z Hasan H Zeid WA. Endovascular management of may-thurner syndrome. Ann Vasc Dis. (2012) 5:217–21. 10.3400/avd.cr.12.00007 23555515 PMC3595863

[9] Patel NH Stookey KR Ketcham DB Cragg AH. Endovascular management of acute extensive iliofemoral deep venous thrombosis caused by May-Thurner syndrome. J Vasc Interv Radiol. (2000) 11:1297–302. 10.1016/S1051-0443(07)61304-9 11099239

[10] Andreozzi GM. Sulodexide in the treatment of chronic venous disease. Am J Cardiovasc Drugs. (2012) 12:73–81. 10.2165/11599360-000000000-00000 22329592

[11] Ulloa JH. Micronized Purified Flavonoid Fraction (MPFF) for patients suffering from chronic venous disease: a review of new evidence. Adv Ther. (2019) 36(Suppl. 1):20–5. 10.1007/s12325-019-0884-4 30758743 PMC6824339

[12] Howlader MH Smith PD. Microangiopathy in chronic venous insufficiency: quantitative assessment by capillary microscopy. Eur J Vasc Endovasc Surg. (2003) 26:325–31. 10.1053/ejvs.2002.1976 14509899

[13] Rabe E Guex JJ Puskas A Scuderi A Fernandez Quesada F Coordinators VCP. Epidemiology of chronic venous disorders in geographically diverse populations: results from the Vein Consult Program. Int Angiol. (2012) 31:105–15. 22466974

[14] Pascarella L Lulic D Penn AH Alsaigh T Lee J Shin H. Mechanisms in experimental venous valve failure and their modification by Daflon 500 mg. Eur J Vasc Endovasc Surg. (2008) 35:102–10. 10.1016/j.ejvs.2007.08.011 17890112

[15] Om H El-Naggar ME El-Banna M Fouda MMG Othman SI Allam AA. Combating atherosclerosis with targeted Diosmin nanoparticles-treated experimental diabetes. Invest New Drugs. (2020) 38:1303–15. 10.1007/s10637-020-00905-6 32048108

[16] Garner RC Garner JV Gregory S Whattam M Calam A Leong D. Comparison of the absorption of micronized (Daflon 500 mg) and nonmicronized 14C-diosmin tablets after oral administration to healthy volunteers by accelerator mass spectrometry and liquid scintillation counting. J Pharm Sci. (2002) 91:32–40. 10.1002/jps.1168 11782895

[17] das Gracas CdSM Cyrino FZ de Carvalho JJ Blanc-Guillemaud V Bouskela E. Protective effects of Micronized Purified Flavonoid Fraction (MPFF) on a novel experimental model of chronic venous hypertension. Eur J Vasc Endovasc Surg. (2018) 55:694–702. 10.1016/j.ejvs.2018.02.009 29588131

[18] Stabile E Burnett MS Watkins C Kinnaird T Bachis A la Sala A. Impaired arteriogenic response to acute hindlimb ischemia in CD4-knockout mice. Circulation. (2003) 108:205–10. 10.1161/01.CIR.0000079225.50817.71 12821542

[19] Shoab SS Porter J Scurr JH Coleridge-Smith PD. Endothelial activation response to oral micronised flavonoid therapy in patients with chronic venous disease–a prospective study. Eur J Vasc Endovasc Surg. (1999) 17:313–8. 10.1053/ejvs.1998.0751 10204053

[20] Shoab SS Scurr JH Coleridge-Smith PD. Plasma VEGF as a marker of therapy in patients with chronic venous disease treated with oral micronised flavonoid fraction - a pilot study. Eur J Vasc Endovasc Surg. (1999) 18:334–8. 10.1053/ejvs.1999.0890 10550269

[21] Zheng Q Pan L Ji Y. H 2S protects against diabetes-accelerated atherosclerosis by preventing the activation of NLRP3 inflammasome. J Biomed Res. (2019) 34:94–102. 10.7555/JBR.33.20190071 32305963 PMC7183300

[22] Dai J Rabie AB. VEGF: an essential mediator of both angiogenesis and endochondral ossification. J Dent Res. (2007) 86:937–50. 10.1177/154405910708601006 17890669

[23] Draker N Torry DS Torry RJ. Placenta growth factor and sFlt-1 as biomarkers in ischemic heart disease and heart failure: a review. Biomark Med. (2019) 13:785–99. 10.2217/bmm-2018-0492 31157982

[24] Ornitz DM Itoh N. Fibroblast growth factors. Genome Biol. (2001) 2:REVIEWS3005. 10.1186/gb-2001-2-3-reviews3005 11276432 PMC138918

[25] Tse E Kwong YL. T-cell lymphoma: microenvironment-related biomarkers. Semin Cancer Biol. (2015) 34:46–51. 10.1016/j.semcancer.2015.06.001 26057207

