# Supplementary figures and images for: Diosmin Alleviates Venous Injury and Muscle Damage in a Mouse Model of Iliac Vein Stenosis
Source: Front Cardiovasc Med. 2022 Jan 13;8:785554. doi: 10.3389/fcvm.2021.785554 (PMC8792538; doi:10.3389/fcvm.2021.785554)

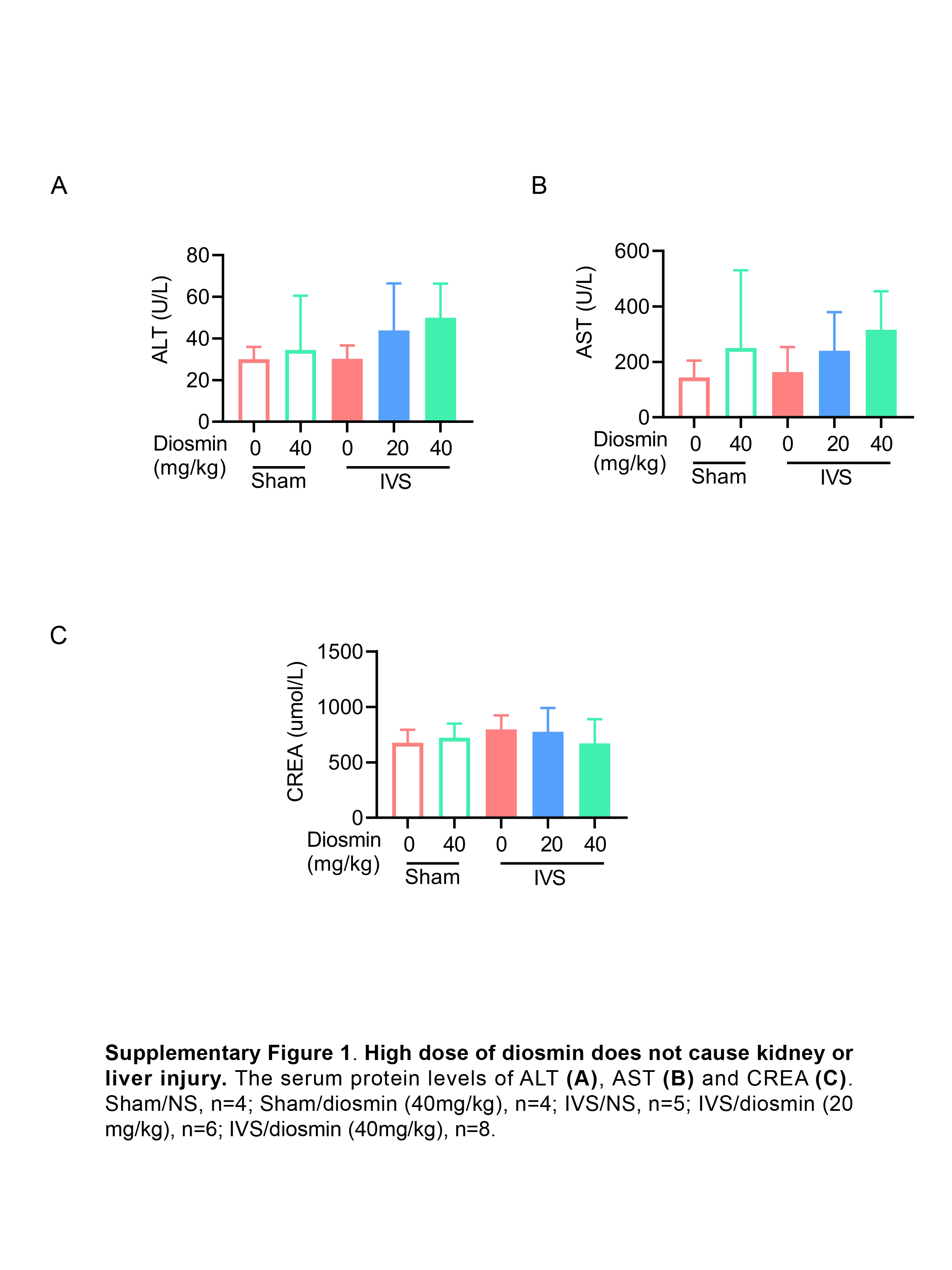

Supplement: Supplementary file 1 [file Image_1.TIF]

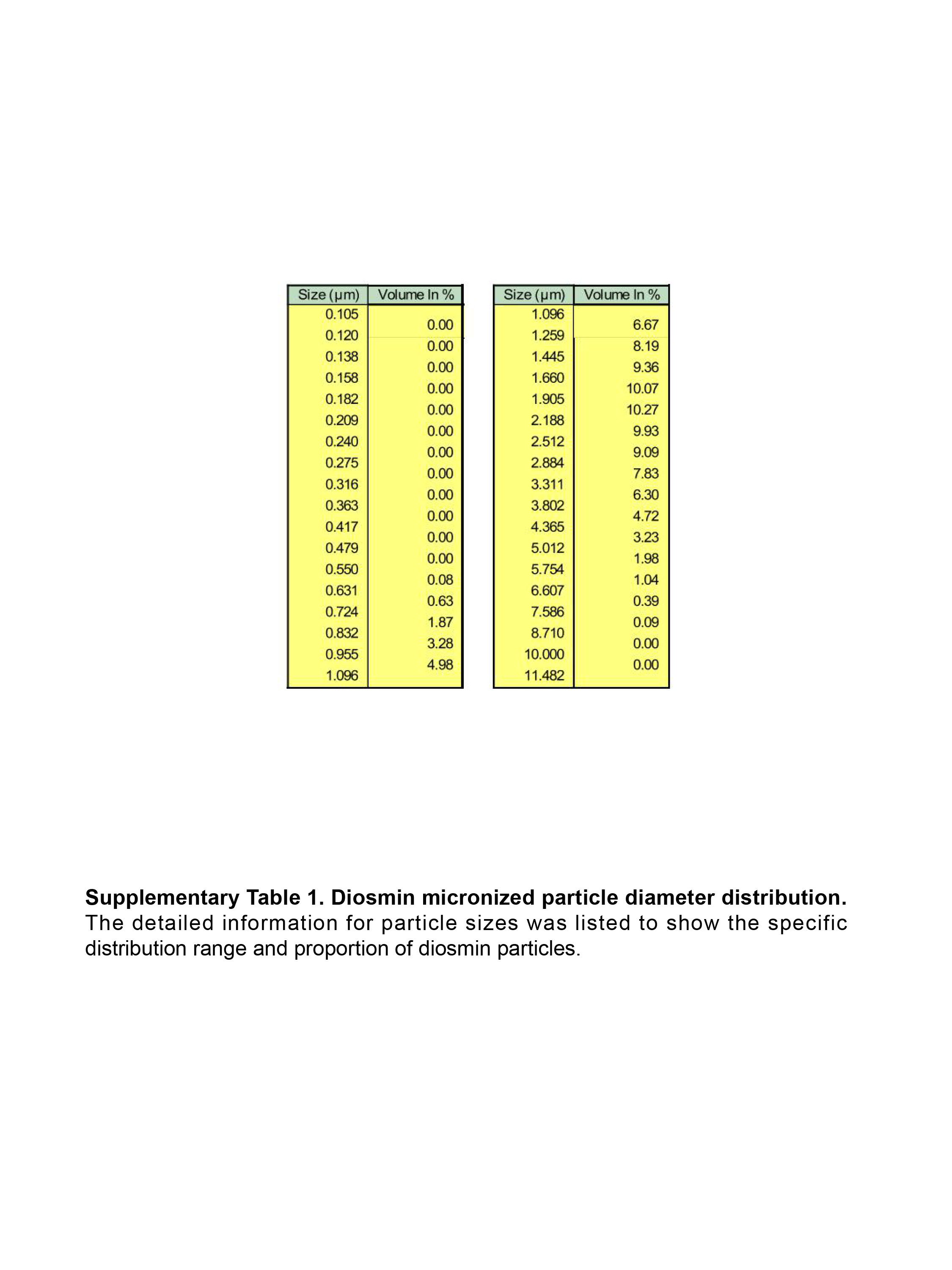

Supplement: Supplementary file 2 [file Image_2.TIF]

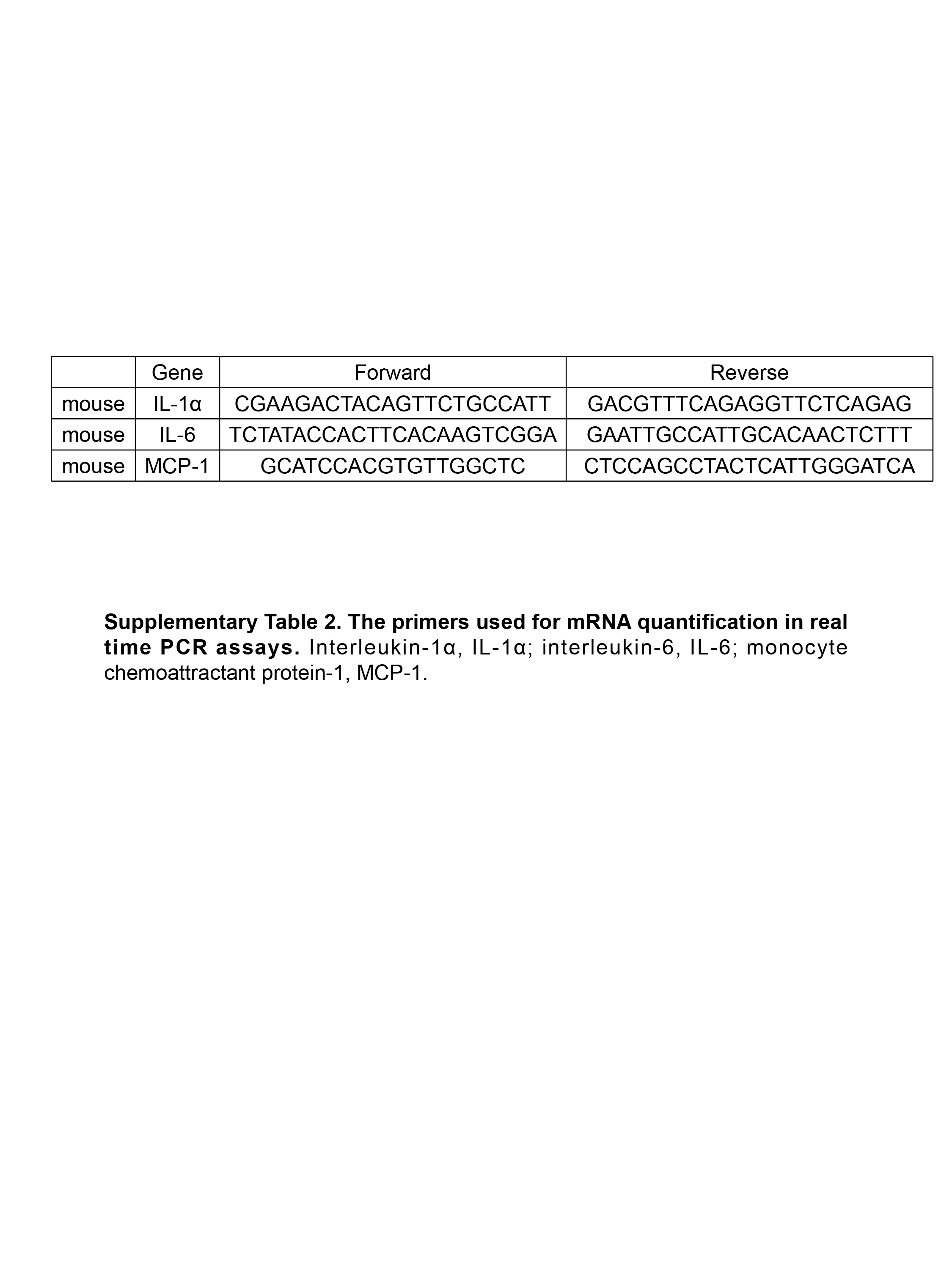

Supplement: Supplementary file 3 [file Image_3.TIF]
